# Supplementary material for: Atorvastatin Augments Gemcitabine-Mediated Anti-Cancer Effects by Inhibiting Yes-Associated Protein in Human Cholangiocarcinoma Cells
Source: Int J Mol Sci. 2020 Oct 14;21(20):7588. doi: 10.3390/ijms21207588 (PMC7589854; doi:10.3390/ijms21207588)
Supplement: Supplementary file 1 [file ijms-21-07588-s001.pdf]

International Journal of
Molecular Sciences

MDPI

Supplementary Materials

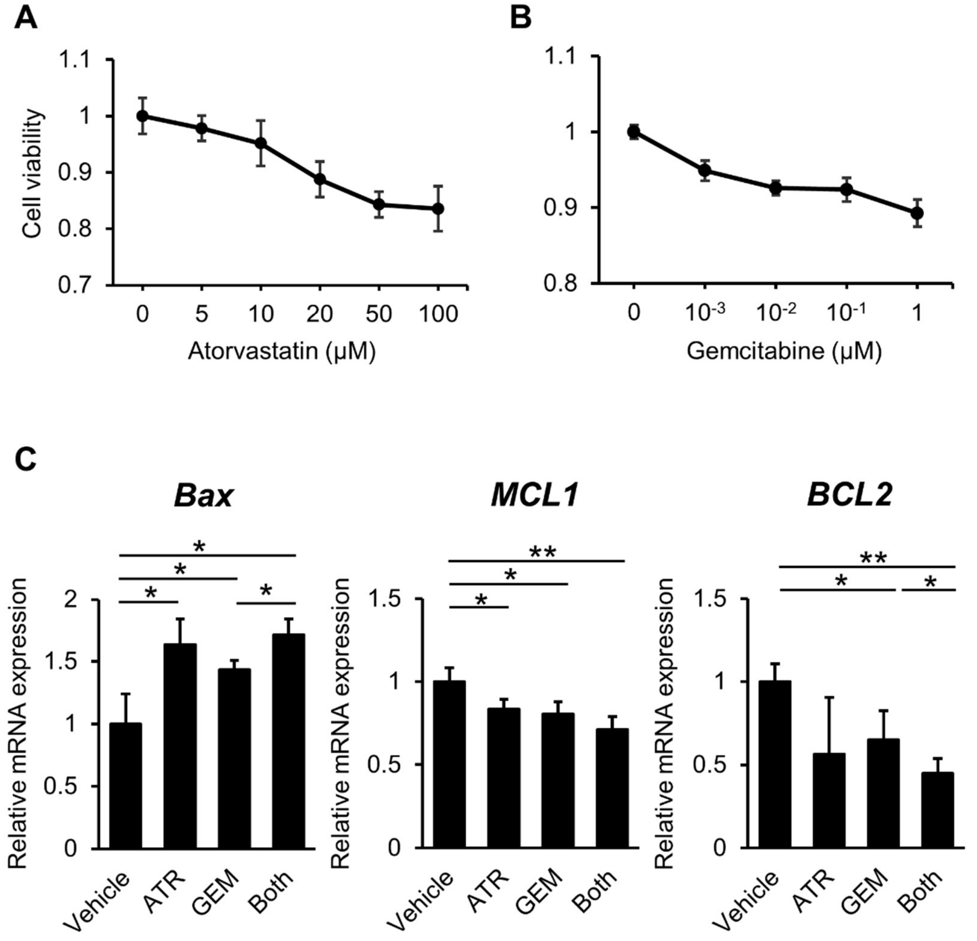

Figure 1. Effects of atorvastatin and gemcitabine on in vitro KKU-M213 cell growth. (**A, B**) Concentration-dependent effects of atorvastatin (0–100 μM) (A) and gemcitabine (0–1 μM) (**B**) on the growth of KKU-M213 cells. (**C**) Relative mRNA levels of BAX, MCL1, and BCL2 in KKU-M213 cells. The mRNA expression levels were measured using quantitative real-time PCR, and glyceraldehyde-3-phosphate dehydrogenase was used as an internal control. Quantitative values are indicated as ratios relative to the values of the vehicle-treated group. ATR, treatment with 20 μM atorvastatin. GEM, treatment with 1×10^{-2} μM gemcitabine. Both, combined treatment with 20 μM atorvastatin and 1×10^{-2} μM gemcitabine. Data are presented as the mean ± SD (n = 10). *, $p < 0.05$; **, $p < 0.01$.

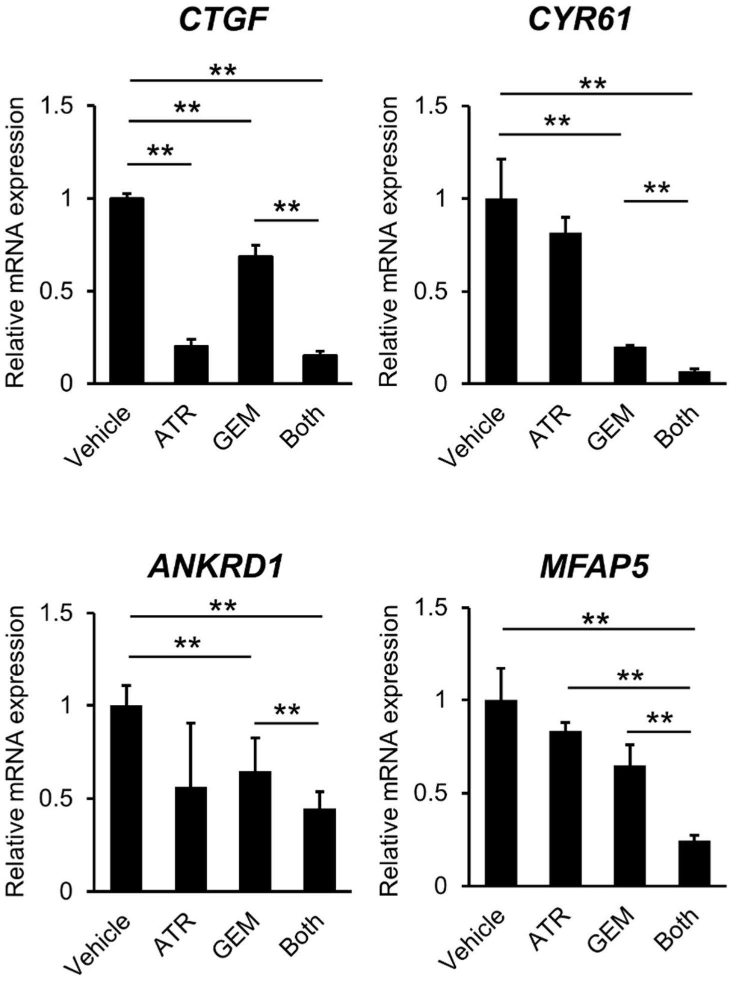

Figure 2. Effects of atorvastatin and gemcitabine on the expression of Yes-associated protein (YAP)/TEA domain (TEAD) target genes in cultured KKU-M213 cells. Relative mRNA levels of the YAP/TEAD target genes CTGF, CYR61, ANKRD1, and MFAP5 in cultured KKU-M213 cells. The mRNA expression levels were measured using quantitative real-time PCR, and glyceraldehyde-3-phosphate dehydrogenase was used as an internal control. Quantitative values are indicated as ratios relative to the values of the vehicle-treated group. ATR, treatment with 20 μM atorvastatin. GEM, treatment with 1×10^{-2} μM gemcitabine. Both, combined treatment with 20 μM atorvastatin and 1×10^{-2} μM gemcitabine. Data are presented as the mean ± SD ($n = 10$). *, $p < 0.05$; **, $p < 0.01$.

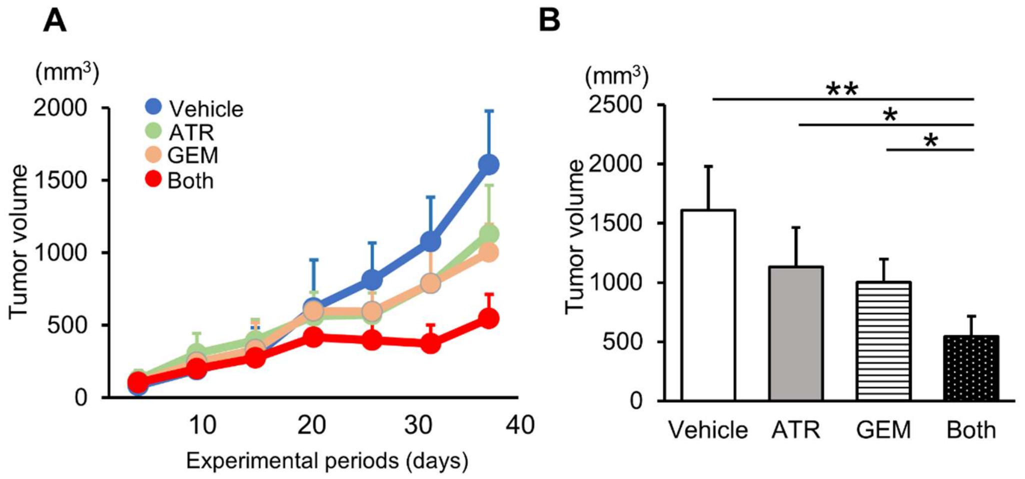

Figure 3. Gemcitabine plus atorvastatin suppressed KKU-M213 cell-derived tumor growth in nude mice. (**A**) KKU-M213 cell-derived xenograft tumors grew progressively in vehicle-treated control mice, but they grew more slowly in mice treated with either atorvastatin (100 mg/kg/day) or gemcitabine (100 mg/kg/3 days). (**B**) The mean tumor volumes in mice treated with atorvastatin and/or gemcitabine. ATR, atorvastatin-treated mice. GEM, gemcitabine-treated mice. Both, mice treated with both atorvastatin and gemcitabine. Data are presented as the mean ± SD (n = 10). *, $p < 0.05$; **, $p < 0.01$.
